# Natural History of Non-Small-Cell Lung Cancer with Bone Metastases

**DOI:** 10.1038/srep18670

**Published:** 2015-12-22

**Authors:** Santini Daniele, Barni Sandro, Intagliata Salvatore, Falcone Alfredo, Ferraù Francesco, Galetta Domenico, Moscetti Luca, La Verde Nicla, Ibrahim Toni, Petrelli Fausto, Vasile Enrico, Ginocchi Laura, Ottaviani Davide, Longo Flavia, Ortega Cinzia, Russo Antonio, Badalamenti Giuseppe, Collovà Elena, Lanzetta Gaetano, Mansueto Giovanni, Adamo Vincenzo, De Marinis Filippo, Maria Antonietta Satolli, Cantile Flavia, Mancuso Andrea, Francesca Maria Tanca, Addeo Raffaele, Russano Marco, M Sterpi, Pantano Francesco, Vincenzi Bruno, Tonini Giuseppe

**Affiliations:** 1 Department of Medical Oncology, University Campus Bio-Medico, Rome, Italy; 2 Division of Medical Oncology, Department of Oncology, Azienda Ospedaliera Treviglio, Piazzale Ospedale 1, 24047, Treviglio, BG, Italy; 3 Unit of Medical Oncology 2, Department of Translational Research and New Technologies in Medicine and Surgery, Azienda Ospedaliero-Universitaria Pisana and University of Pisa, Via Roma 67, 56126 Pisa, Italy; 4 Division of Medical Oncology, S.Vincenzo Hospital, Taormina, Italy; 5 Medical Oncology Unit, Clinical Cancer Center, Istituto Tumori “Giovanni Paolo II”, Viale Orazio Flacco 65, 70124 Bari, Italy; 6 Department of Oncology and Hematology, ‘Belcolle’ Hospital, Viterbo, Italy; 7 Department of Oncology, A.O. Fatebenefratelli e Oftalmico, Corso di Porta Nuova 23, 20121, Milan, Italy; 8 Osteoncology and Rare Tumors Center, Istituto Scientifico Romagnolo per lo Studio e la Cura dei Tumori (IRST) IRCCS, Meldola (FC), Italy; 9 Department of Medical Oncology, Presidio Sanitario Gradenigo, Turin, Italy; 10 Department of Molecular Medicine, University Sapienza, Rome, Italy; 11 Department of Medical Oncology, Institute for Cancer Research & Treatment (IRCC), Candiolo, Torino, Italy; 12 Department of Surgery and Oncology, Section of Medical Oncology, University of Palermo, Palermo, Italy; 13 Medical Oncology, AO Ospedale Civile di Legnano, Italy; 14 Istituto Neurotraumatologico Italiano, Unità Funzionale di Oncologia, Grottaferrata, Italy; 15 Medical Oncology Department, General Hospital, Frosinone, Italy; 16 Department of Human Pathology, University of Messina, Messina, Italy ; Medical Oncology Unit, Azienda Ospedaliera Ospedali Riuniti Papardo-Piemonte, Messina, Italy; 17 Division of Thoracic Oncology, European Institute of Oncology (IEO), Milan, Italy; 18 Department of Oncology, University of Torino, Torino, Italy; 19 Dipartimento di Patologia Generale , Facoltà di Medicina e Chirurgia Seconda Università degli studi, Naples, Italy; 20 Department of Medical Oncology, San Camillo and Forlanini Hospitals Rome, Italy; 21 Department of Medical Oncology, University of Cagliari, Cagliari, Italy; 22 U.O. Oncologia, ASL NA2 NORD, Frattamaggiore, Italy

## Abstract

We conducted a large, multicenter, retrospective survey aimed to explore the impact of tumor bone involvement in Non-Small Cell Lung Cancer.Data on clinical-pathology, skeletal outcomes and bone-directed therapies for 661 deceased patients with evidence of bone metastasis were collected and statistically analyzed. Bone metastases were evident at diagnosis in 57.5% of patients. In the remaining cases median time to bone metastases appearance was 9 months. Biphosphonates were administered in 59.6% of patients. Skeletal-related events were experienced by 57.7% of patients; the most common was the need for radiotherapy. Median time to first skeletal-related event was 6 months. Median survival after bone metastases diagnosis was 9.5 months and after the first skeletal-related event was 7 months. We created a score based on four factors used to predict the overall survival from the diagnosis of bone metastases: age >65 years, non-adenocarcinoma histology, ECOG Performance Status >2, concomitant presence of visceral metastases at the bone metastases diagnosis. The presence of more than two of these factors is associated with a worse prognosis.This study demonstrates that patients affected by Non-Small Cell Lung Cancer with bone metastases represent a heterogeneous population in terms of risk of skeletal events and survival.

Lung cancer is the most common solid tumor and the leading cause of human cancer deaths worldwide^1^.

In the last decades the introduction of platinum-based chemotherapy, of third-generation cytotoxic drugs (such as gemcitabine, vinorelbine, docetaxel and pemetrexed), of monoclonal antibodies (such as Bevacizumab), and of novel targeted therapies has radically modified the treatment of advanced NSCLC. Consequently, median overall survival (OS) for patients with advanced lung cancer has increased from approximately 6 months to 12 months, and is longer for patients with driver mutations treated with targeted therapies^2^,^3^.

Nevertheless, the prognosis of NSCLC patients remains poor. In fact, the majority of cases is diagnosed at metastatic-stage.

NSCLC is the third most common cause of bone metastases (following breast and prostate cancer). The incidence of bone metastasis in this kind of tumor during the clinical course of the disease is 30–40%^4^,^5^, and 60% of these patients presents bone metastasis at the time of diagnosis^6^. Twenty years ago it was reported that the median survival time (MST) of patients with bone metastases was 7 months^7^. Moreover, survival improvement in patients affected by NSCLC may have determined a further increase in the incidence of bone metastases and in all likelihood a change in the natural history of this disease. On the other hand, the presence of bone metastases itself seems to represent a negative prognostic factor for patients affected by NSCLC^8^. It is clear that in this setting, bone metastases diagnosis and treatment become a relevant clinical problem. Scientific evidence shows that bone metastases have a greater negative impact on the OS and the quality of life of patients affected by solid tumours^9^,^10^. Indeed, bone lesions are often complicated by SREs such as: radiotherapy, pathological fractures, spinal cord compression, orthopaedic surgery and hypercalcemia. SREs cause pain and decreased quality of life, with declines in physical, functional and emotional well being and negatively affect survival^11^,^12^.

The aim of this retrospective multicenter survey was to evaluate the natural history of skeletal disease in NSCLC patients, to detect the impact of bone metastases on the outcome of the disease and to examine the role of several clinical-pathological parameters in predicting survival in these patients. Similar studies have been conducted, above all for solid tumours such as breast, prostate, kidney and colorectal cancer. On the contrary, the data about NSCLC available in literature are poor, of ambiguous interpretation and only based on small cohorts of patients.

## Patients and Methods

### Ethics Statement

The Ethics Committee of the coordination center has approved this multicenter retrospective observational study. Furthermore the Ethics Committee deemed unnecessary a written consent in consideration of the fact that the data the study was built on were related to patients already dead by the time it was conducted and therefore their treatment was in no way impacted or influenced by it.

The methods were carried out in accordance with the approved guidelines.

### Study design

This retrospective, observational multicenter study included consecutive NSCLC patients with bone metastasis and was conducted in 18 Italian hospital centers in which these patients received their diagnosis and treatment from 1999 to 2012.

The data utilized pertained to NSCLC patients of all ages who were never enrolled in any clinical trials or experimental protocols and whose treatment followed the practice adopted by their respective physician. These patients were diagnosed with at least one bone metastasis during the course of their disease and their death was caused by NSCLC or cancer-related complications. More specifically, in order to be identified as having bone metastasis, at least two of the following criteria had to be fulfilled: the presence of bone metastasis was reported by the physician; the identification of bone metastasis was made by body scan; a record proved the use of radiotherapy for the bone for palliative purposes; bone metastasis was detected by other imaging assessment (e.g. standard x-rays, computed tomography scans, or magnetic resonance imaging of the skeleton). The data related to each patient covers the whole course of the disease and all cancer treatments, including surgery, radiation therapy, chemotherapy and biological therapies. Assessed variables included age, histotype, number and sites of bone metastases, time to appearance of bone metastasis, times to first and subsequent SREs (from diagnosis of bone metastasis), SRE types, survival after the first SRE, survival after bone metastasis diagnosis, type and times of bisphosphonate therapy.

### Statistical analysis

The incidence of SREs and patient demographics were determined through descriptive statistics and the Kaplan-Meier method was used to estimate all survival intervals^13^. The Kaplan- Meier method was also adopted in order to describe the differences in survival that were compared through the use of the log-rank test in accordance with clinical variables or treatment^14^. All the clinical parameters were evaluated as possible predictor factors for shorter time to bone metastasis, shorter time from bone metastases to SRE and shorter time from bone metastases to death by an univariate model in which all patients without a record of the date of a specific event were left censored at the date of death.

All the variables found to be significant in the univariate model were then employed in the multivariate survival analysis that was assessed by the application of the Cox proportional hazards model. Instead of using values deriving from fractions of a month, the median values were calculated from whole-month values. Finally, statistical analysis was conducted through an SPSS software (version 20.00; SPSS, Chicago, IL). To be considered statistically significant, the *p* value had to be under 0.05.

## Results

### Patient characteristics

After screening of more than 2000 patients, died from NSCLC, we identified 661 patients with bone metastasis (33%).

The median age at NSCLC diagnosis was 64 years old, range 22 to 88. Adenocarcinoma represented the most common histotype (69.3%). At the time of lung cancer diagnosis, only 20 patients had stage I disease (3.1%), 24 patients had stage II (3.7%), 48 (7.3%) stage IIIa, 45 (6.9%) IIIb and 517 (79%) stage IV. 85% of patients, at the time of diagnosis, had an ECOG PS 0–1.

The EGFR mutational analysis was unknown in 70.4% of cases; in the other 29.4% of patients, with known EGFR analysis, 74.9% was EGFR wild type and 25.1% was mutated. 18.6% of the patients went under surgical resection of primitive tumour. Most of the enrolled patients received a first-line treatment (91.7%): chemotherapy was preferred in 94.3% of patients (in the 59.4% of cases a chemotherapeutic schedule based on platinum was chosen). In 30.6% of patients, a thirosyne-kinase inhibitor (TKI) was used (mostly in subsequent lines): gefitinib in the 22.1% of cases and erlotinib in the 77.9%. [Table 1].

### Skeletal metastases and SREs

In the analyzed sample, 83,8% of patients had visceral metastasis. 57.5% of patients presented bone metastasis at the time of diagnosis. In the remaining cases, the median time to bone metastasis onset was about 9 months (range 1–73 months).

The median time to diagnosis of bone metastases since diagnosis of NSCLC in non-metastatic patients was 19 months in patients at stage I, 21 months in patients at stage II, 12 months at stage IIIa and 10 months in patients at stage IIIb.

At the time of the diagnosis of bone metastasis 50 patients had an ECOG performance status 0 (23.1% of cases); 339 patients (52.2%) had an ECOG PS 1, 134 (20.6%) ECOG PS 2 and 27 (4.2%) ECOG PS 3 (median ECOG PS was 1). In the 78.3% of cases, patients had multiple metastasis and 74.3% of them were osteolytic lesions; 11.4% osteoblastic and 14.3% of cases had mixed lesions. The axis was involved in 74,9% of cases, pelvic bones in 48.1%, limbs in 32.9%; other sites in 34.5%. 78% of patients reported bone pain at the time of bone metastasis diagnosis. The median Verbal Numerical Rating Scale (VNRS) value for pain was 4; pain value ≥4 was referred in the 44.3% of cases. Nevertheless skeletal pain was registered during disease course in all the patients; the median value of maximum pain was 7 (at the time of diagnosis 79.2% of patients had a VNRS value ≥4).

At least one SRE characterized the clinical history of 57.7% of patients out of which 42.5% had a single SRE, 11.9% had two SREs and only the 3% experimented at least 3 SREs. The most common first, second and third SRE was represented by the need of radiotherapy (in the 71.4%, 79.2% and 61.9% of cases, respectively); followed by pathologic fractures: in the 16.3%, 9.4% e 19% of SREs cases respectively [Table 2].

The median time to the first SRE was 6 months (range 0–57 months). 59.6% of patients received bisphosphonates. Zoledronic acid was the most used bisphosphonate (56.2% of all patients) and its administration preceded the first SRE in the 33.4% of cases. 1.4% of cases (9 patients among 661) reported osteonecrosis of the jaw (ONJ).

On Tables 3, 4, 5 we reported all parameters that showed a correlation with the time to bone metastases onset and overall survival since skeletal metastasis diagnosis, respectively in univariate and multivariate analyses. In the multivariate analysis only two parameters (advanced stage and lack of surgery) were correlated with an earlier occurrence of bone metastasis. In the multivariate analysis regarding overall survival since diagnosis of bone metastases, only five parameters remained statistically significant: histology, stage at diagnosis, platinum-based chemotherapy, the concomitant presence of visceral metastases and the use of Zoledronic acid before the first SRE onset [Tables 3, 4, 5].

### Outcomes

The median overall survival from bone metastasis diagnosis was 9.5 months and the median overall survival from the first SRE was 7 months.

The median survival time in patients without SREs was 8 months; on the other hand, in patients who experimented at least one SRE was 10 months. Nevertheless, selecting the patients with an SRE as onset of bone disease, the prognosis appears to be worse than in patients who develop an SRE after diagnosis of metastases bones (OS was 6 months versus 10 months, p 0,017).

The median time between bone metastasis diagnosis and the first SRE was 14 months in biphosphonates treated patients and 7 months in patients who had not received bisphosphonates before the first SRE. The median survival from bone metastasis in patients treated with biphosphonates was 9 months and in patients that did not receive treatment was 5 months. In both cases the differences were statistically significant (p 0.01).

The concomitant presence of visceral metastases seems to be associated with a worse prognosis. The median overall survival from diagnosis of bones metastases in patients with concomitant visceral metastases was 7-months versus 10 months in patients without metastases (univariate p 0.001; multivariate P value 0.002-HR:1.354 95% C.I : 1.114-1,647).

**Figure 1:**
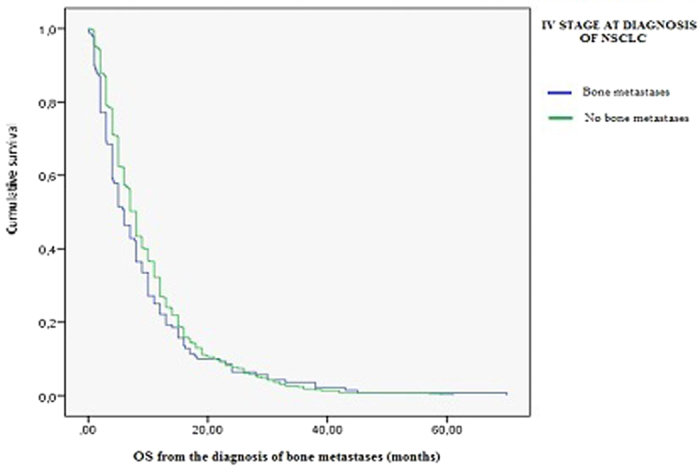
IV stage at diagnosis: patients with or without bone metastases. Kaplan-Meier survival analysis.

The selective evaluation of patients with stage IV at diagnosis of NSCLC has not shown statistically significant differences in OS between patients with bone metastases and patients without bone metastases at diagnosis [Fig. 1]. Not even the time to the onset of bone metastases appears to be a factor able to predict differences in overall survival from diagnosis of bone metastases (Cox regression model; P value 0,172).

**Figure 2:**
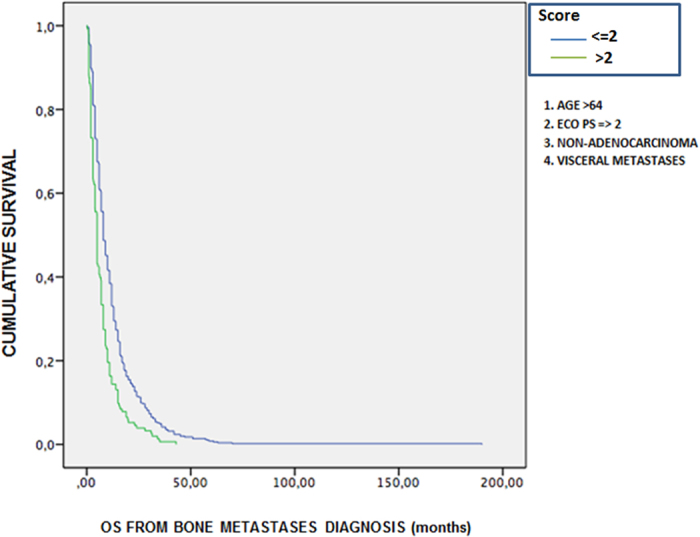
Score to predict a different prognosis at diagnosis of bone metastases. Kaplan-Meier survival analysis.

Finally, we created a score based on four factors that were found to be significant in the univariate analysis used to predict the overall survival from the diagnosis of bone metastases: age >65 years, non-adenocarcinoma histology, ECOG Performance Status >2, concomitant presence of visceral metastases at the bone metastases diagnosis. According to this score, the presence of more than two of these four factors is associated with a worse prognosis: median survival was 5 months versus 8 months of the other group of patients (P value < 0,001) [Fig. 2].

## Discussion

This work has the advantage of providing data from a large cohort of patients. To our knowledge, this study is the largest multicenter survey investigating the natural history of metastatic bone disease in patients with NSCLC. The high number of patients and the collection of many clinical and therapeutic parameters have enabled us to identify for each of them the correlation with the quality of life and the prognosis of these patients.

Data concerning NSCLC epidemiology are coherent with the ones found in literature such as major incidence in later age, adenocarcinoma as prevalent histotype, diagnosis of lung cancer more frequent at metastatic stage.

Patients with bone metastases treated with TKIs or Bevacizumab as first-line treatment had better survival rates compared with those treated with only chemotherapy. This finding confirms and emphasizes the use of biological drugs in this setting of patients.

Clinical data show that pain is the most observed symptom, affecting almost every patient at the moment of bone metastasis diagnosis, and all patients during the clinical course of the disease. Considering bone pain and performance status (which is worsened by the onset of bone metastases in approximately 17% of patients) as parameters for measuring the quality of life, we can assume that skeletal metastases significantly impact the quality of life of the patients. Our study did not evaluate the impact of the analgesic therapy, although it is clear that an adequate pain management is extremely important for these patients. Specific studies on this matter are needed to identify the correct management of bone pain in patients with NSCLC.

Bone metastases are more frequently multiple and osteolytic and can affect any segment of the skeleton, mainly the spine followed by long and pelvic bones. Skeletal complications occur in more than half of the cases. Radiation therapy is the most common SRE since it is one of the main tools to relieve pain which is the predominant symptom in these patients. Hypercalcemia, according to other studies, is reported in 12–20% of patients with lung cancer^15^. In our study it is present in a lower percentage (3%, 20/661 patients). This discrepancy could be due to the fact that the evidence already present in literature reports the incidence of hypercalcemia in all patients with lung cancer, whereas in our study we enrolled only patients with NSCLC and bone metastases treated mostly with bisphosphonates.

The median survival time in patients without SREs was lower than in patients who experimented at least one SRE therefore it seems plausible to assume that the presence of skeletal events is a favorable prognostic factor. This finding is in disagreement with similar analysis conducted for other solid tumors, including studies regarding Lung Cancer; however these analyses were carried on a much lower number of patients compared to our study^15^. Our hypothesis is that this inconsistency can be explained by the prolonged survival of patients with NSCLC registered in recent years. Namely, patients who live longer have a greater chance of developing SRES.

Conversely, patients with an SRE as onset of bone disease have a worse prognosis than those who develop an SRE after diagnosis of metastases bones. These data would suggest the possible existence of two different types of skeletal disease secondary to NSCLC: one particularly aggressive where the presence of SRE (especially if manifested as onset of bone disease) is associated with a worse prognosis, and an other, less aggressive, in which increased survival is associated with an increased risk of developing SREs.

Bisphosphonates were proven to delay by 7 months the first SRE after diagnosis of bone metastases compared to untreated patients (14 vs. 7 months) (P value 0,003). Regarding survival rates, bisphosphonates appear to correlate with increased survival (9 vs 5 months) (P value 0,006). However, this data may be the result of a selection bias, since bisphosphonates are administered to patients with good life expectancy and also a consequence of the retrospective nature of this analysis.

The role of these agents in the treatment of advanced NSCLC has not been investigated in a large phase III trial. Neverthless, Rosen *et al.*^16^ showed the long-term efficacy of zoledronic acid in the treatment of skeletal metastasis in patients with NSCLC. The results of our study would confirm these findings and would encourage the use of bisphosphonates in this setting of patients. Furthermore, denosumab is a new bone-targeted therapy. Its efficacy is documented in patients with bone metastases arising from solid tumors (including NSCLC)^17^. Patients included in our study did not receive denosumab because they were retrospectively enrolled before this drug started to be used in this setting.

Limitations of our study include its retrospective design and inclusion of an unselected heterogeneous cohort of patients with all types of histological (squamous, adenocarcinoma, large cell, undifferentiated) variants of NSCLC, molecular subclassifications (EGFR, ALK, ROS-1, etc) as well as a wide range of anticancer therapies. However, the types of patients included in this study represent the typical scenario of real clinical practice. Another limitation is the heterogeneity of standardized methods used for detecting bone metastases, with each methodology having its own limit of detection.

Nonetheless, we believe that this study will enhance the knowledge of the natural history of NSCLC with bone metastases and will aid to identify some parameters that have a real prognostic value in this setting of patients. Furthermore, it has helped in providing a score that may be used in clinical practice, if validated in other pools of patients, to recognize patients with different outcomes at the time of skeletal disease diagnosis.

Further studies, especially prospective ones regarding the treatment of bone pain and specific treatments of bone metastases (bisphosphonates, denosumab, radiotherapy) are required and could offer interesting perspectives on how to improve not only the quality of life but also the survival of patients affected by NSCLC with bone metastases.

## Additional Information

**How to cite this article**: Daniele, S. *et al.* Natural History of Non-Small-Cell Lung Cancer with Bone Metastases. *Sci. Rep.* **5**, 18670; doi: 10.1038/srep18670 (2015).

## Figures and Tables

**Table 1:** Tumor characteristics and treatment.

Tumor Characteristics And Treatments	N° Patients
Histotype	
Adenocarcinoma	69,3% (436)
Other histotypes	30,7% (198)
Stage	
I	3,1% (20)
II	3,7% (24)
IIIa	7,3% (48)
IIIb	6,9% (45)
IV	79% (517)
Egfr Mutation	
Unknown	70,5% (459)
Known	29,5% (195)
Wild Type	74,9% (146)
Mutated	25,1% (49)
Surgery	
No	81,4% (531)
Yes	18,6% (121)
First-Line Treatment	
Chemotherapy	
No	5,7% (34)
Yes	94,3% (564)
Platinum-based	54,9% (388)
Other Therapies	45,1% (265)
Tkis	
No	69.4% (452)
Yes	30,6% (199)
Gefitinib	22,1% (44)
Erlotinib	77,9% (155)

**Table 2:** Most frequent first, second, third and subsequent SREs.

SREs	First SRE	Second SRE	Third and subsequent SREs
Radiotherapy	71.4% (262)	79.2% (76)	61.9% (13)
Pathologic fractures	16.3% (60)	9.4% (9)	19% (4)
Spinal cord compression	6% (22)	2.1% (2)	9.5% (2)
Hypercalcemia	4.1% (15)	4.2% (4)	9.5% (2)
Surgery	3.3% (12)	5.2% (5)	14.3% (3)

**Table 3:** Time to first bone metastasis onset.

Univariate Analysis				
Parameters		Median Time to bone met (months)	P-value	95% CI
Age	>64	5	0.046	3.021–6.979
	<64	7		5.503–8.497
ECOG PS at diagnosis	0–1	7	0.012	5.928–8.072
	>2	2		0.000–4.191
Stage at diagnosis	I	16	0.001	9.426–22.574
	II	19		2.197–35.803
	IIIa	12		9.739–14.261
	IIIb	7		4.863–9.137
	IV	4		3.363–4.637
Surgical resection	Yes	11	0.004	6.051–15.949
	No	6		4.788–7.212
First-line treatment	CT	6	0.008	5.142–6.858
	TKIs	12		4.160–19.840
Pelvic bone metasta	Yes	4.2	0.023	2.495–5.905
sis	No	8		5.835–10.165
Limb bone mestastasis	Yes	5	0.019	2.326–7.674
	No	7		5.447–8.553

Parameters which showed statistical significativity in the univariate analysis.

**Table 4:** Overall survival from bone metastasis diagnosis.

Univariate Analysis				
Parameters		Median OS (months)	P-value	95% CI
Age	>64	7	0.008	6.253–7.747
	<64	8		7.161–8.839
ECOG PS at diagnosis	0–1	8	0.001	7.457–8.543
	>2	3.5		3.080–3.920
Histology	Adenocarcinoma	8	0.001	7.099–8.901
	Others	6		5.312–6.688
Stage at diagnosis	I	14	0.004	9.639–18.631
	II	6		2.412–9.588
	IIIa	9		7.075–10.925
	IIIb	9		5.720–12.280
	IV	7		6.437–7.563
First-line treatment	CT	8	0.001	7.463–8.537
	TKIs	3		2.324–3.676
Platinum-based chemotherapy	Yes	8	0.001	7.081–8.919
	No	5		4.089–5.911
First-line TKIs	Yes	12	0.001	10.466–13.534
	No	6		5.395–6.605
ECOG PS at bone metastasis diagnosis	0–1	8	0.001	7.510–8.490
	>2	4		3.104–4.896
Number of SREs	0	6	0.001	5.403–6.597
	1	8		7.117–8.883
	2	10		7.330–12.670
	3	12		7.268–17.932
Pathologic fracture	Yes	7	0.040	5.026–8.974
	No	8		6.744–9.256
Spinal cord compression	Yes	7	0.008	4.740–9.260
	No	9		7.864–10.136
Use of Biphospho	Yes	9	0.001	8.046–9.954
nates	No	5		4.244–5.756
Use of Zoledronic acid	Yes	9	0.001	8.120–9.880
	No	5		4.202–5.798
Use of Zoledronic acid before the first SRE onset	Yes	10	0.001	8.594–11.406
	No	7		6.358–7.642
Concomitant presence of visceral metastases	*Yes*	7	0.001	6.383–7.617
	*No*	10		8.277–11.722

Parameters which showed statistical significativity in the univariate analysis.

**Table 5:** Overall survival from bone metastasis diagnosis.

Multivariate Analysis					
Parameters		Median OS (months)	HR	P-value	95% CI
*Histology*	*Adenocarcinoma*	8	1,296	0.049	1.001–1.677
	*Others*	6			
*Stage at diagnosis*	*I*	14	1,17	0,01	1.039–1.327
	*II*	6			
	*IIIa*	9			
	*IIIb*	9			
	*IV*	7			
*Platinum-based chemotherapy*	*Yes*	8	0,66	0.002	0.511–0.861
	*No*	5			
Use of Zoledronic acid before the first SRE onset	*Yes*	10	0,77	0,046	0,609–0,995
	*No*	7			
Concomitant presence of visceral metastases	*Yes*	7	1.354	0.002	1.114–1,647
	*No*	10			

Parameters which showed statistical significativity in the multivariate analysis.
