# Corrigendum: Natural History of Non-Small-Cell Lung Cancer with Bone Metastases

**DOI:** 10.1038/srep22205

**Published:** 2016-04-15

**Authors:** Daniele Santini, Sandro Barni, Salvatore Intagliata, Alfredo Falcone, Francesco Ferraù, Domenico Galetta, Luca Moscetti, Nicla La Verde, Toni Ibrahim, Fausto Petrelli, Enrico Vasile, Laura Ginocchi, Davide Ottaviani, Flavia Longo, Cinzia Ortega, Antonio Russo, Giuseppe Badalamenti, Elena Collovà, Gaetano Lanzetta, Giovanni Mansueto, Vincenzo Adamo, Filippo De Marinis, Maria Antonietta Satolli, Flavia Cantile, Andrea Mancuso, Francesca Maria Tanca, Raffaele Addeo, Marco Russano, Michelle Sterpi, Francesco Pantano, Bruno Vincenzi, Giuseppe Tonini

Scientific Reports 5: Article number: 18670; 10.1038/srep18670 published online: 12222015; updated: 04152016.

The original version of this Article contained errors in the spelling of the authors Daniele Santini, Sandro Barni, Salvatore Intagliata, Alfredo Falcone, Francesco Ferraù, Domenico Galetta, Luca Moscetti, Nicla La Verde, Toni Ibrahim, Fausto Petrelli, Enrico Vasile, Laura Ginocchi, Davide Ottaviani, Flavia Longo, Cinzia Ortega, Antonio Russo, Giuseppe Badalamenti, Elena Collovà, Gaetano Lanzetta, Giovanni Mansueto, Vincenzo Adamo, Filippo De Marinis, Flavia Cantile, Andrea Mancuso, Raffaele Addeo, Marco Russano, Michelle Sterpi, Francesco Pantano, Bruno Vincenzi and Giuseppe Tonini which were incorrectly given as Santini Daniele, Barni Sandro, Intagliata Salvatore, Falcone Alfredo, Ferraù Francesco, Galetta Domenico, Moscetti Luca, La Verde Nicla, Ibrahim Toni, Petrelli Fausto, Vasile Enrico, Ginocchi Laura, Ottaviani Davide, Longo Flavia, Ortega Cinzia, Russo Antonio, Badalamenti Giuseppe, Collovà Elena, Lanzetta Gaetano, Mansueto Giovanni, Adamo Vincenzo, De Marinis Filippo, Cantile Flavia, Mancuso Andrea, Addeo Raffaele, Russano Marco, M Sterpi, Pantano Francesco, Vincenzi Bruno and Tonini Giuseppe respectively.

The Author Contributions Statement,

S.D., I.S. and R.M. wrote the main manuscript. R.M. and P.F. prepared tables and figures. S.D., B.S., I.S., F.A., F.F., G.D., M.L., L.V.N., I.T., P.F., V.E., G.L., O.D., L.F., O.C., R.A., B.G., C.E., L.G., M.G., A.V., D.M.F., S.M.A., C.F., M.A., T.F.M., A.R., R.M., P.F., V.B. and T.G. made substantial contributions to concept and design of the study, revised the manuscript and gave their approval to the final version of the manuscript.

now reads

D.S., S.I. and M.R. wrote the main manuscript. M.R. and F.P. prepared tables and figures. D.S., S.B., S.I., A.F., F.F., D.G., L.M., N.L.V., T.I., F.P., E.V., L.G., D.O., F.L., C.O., A.R., G.B., E.C., G.L., G.M., V.A., F.D.M., M.A.S., F.C., A.M., F.M.T., R.A., M.R., F.P., B.V. and G.T. made substantial contributions to concept and design of the study, revised the manuscript and gave their approval to the final version of the manuscript.

In addition, the ‘How to cite this article’ section quoted an incorrect abbreviation for Daniele Santini.

These errors have now been corrected in the PDF and HTML versions of the Article.

